# A New Suppression Index Calculation Using the Visually Enhanced Vestibulo-Ocular Reflex and Vestibulo-Ocular Reflex Suppression Paradigms in the Video Head Impulse Test

**DOI:** 10.3390/audiolres14040063

**Published:** 2024-08-22

**Authors:** Carlos Prieto-Matos, Jorge Rey-Martínez, Nicolás Pérez-Fernández

**Affiliations:** 1 ENT Department, Clínica Universidad de Navarra, 31008 Pamplona, Spain; 2 Neurotology Unit, ENT Department, Hospital Universitario Donostia, 20014 Donostia-San Sebastián, Spain; 3 ENT Department, Clínica Universidad de Navarra, 28027 Madrid, Spain; nperezfer@unav.es

**Keywords:** VORS, VOR, suppression index, clinical test, quantification, gain

## Abstract

The aim of this study is to calculate the gains of the quantified visually enhanced vestibulo-ocular reflex (qVVOR) and the quantified vestibulo-ocular reflex suppression (qVORS), using a specific system to generate a visual suppression index (SI) in healthy subjects obtained through the gains of qVVOR and qVORS, and to determine the normal values of the index, as well as the influence of age and sex variables on the SI. Methods. This prospective observational clinical study includes 83 healthy subjects who underwent the head impulse and suppression tests (HIMP and SHIMP, respectively), qVVOR, and qVORS tests, all of the vHIT. The sinusoidal tests (qVVOR and qVORS) were conducted at an intended frequency of 0.75 Hz. The gains of these tests were calculated using a system specifically designed for this purpose. A formula for the SI was established using a ratio of the gains from these tests. Two SI values are presented: unilateral, distinct for each direction of head movement, and bilateral, representing the suppression of both sides simultaneously. Results. Mean gains for the qVVORs were 0.981 ± 0.070 and 0.978 ± 0.077 for the rightwards and leftwards qVVORs, respectively. The gains for the suppressed tests were 0.334 ± 0.112 and 0.353 ± 0.110 for the rightwards and leftwards qVORSs, respectively. A difference of 0.05 Hz was observed between the expected (0.75 Hz) and the obtained frequency of head movement, which is statistically significant (*p* < 0.001). The SI was 0.342 ± 0.118 for the right side (right SI) and 0.363 ± 0.117 for the left side (left SI). The bilateral SI had a mean value of 0.295 ± 0.104. No significant differences in the SI were noted according to the subject’s age. The SI for women was lower than in the case of males. Conclusions: The VVOR/VORS quantification algorithm allows for the reliable calculation of the numerical gain of qVVOR and qVORS with mathematical soundness and consistency of results. Our data support the use of a single or specific measure for direction of head movement; although significant differences exist, these differences are not clinically relevant.

## 1. Introduction

The visually enhanced vestibulo-ocular reflex (VVOR) and the vestibulo-ocular reflex suppression (VORS) tests, first clinically characterized by Halmagyi in 1979, can now be performed using the video head impulse test (vHIT) system for greater sensitivity [1]. In the VVOR test, subjects perform slow, sinusoidal head movements while maintaining gaze on a stationary point, resulting in stable eye positioning mediated by a slow eye movement in the opposite direction to the head at the same velocity without refixation saccades [2]. VOR suppression, mediated by cognitive circuits for gaze stabilization, is executed by having the fixation point move with the head, such as by fixing the gaze on an extended thumb during head movements. In healthy patients, the eyes move, resulting also in a stable retinal image [3,4,5,6].

These tests primarily explore the VOR, with low-frequency sinusoidal movements activating additional central gaze-holding systems like smooth pursuit (SP) and the optokinetic reflex (OKN) [7,8]. An abnormal VORS can indicate central dysfunction [3,9]. When high-frequency stimuli (>1 Hz) are applied, only the VOR is active; for low-frequency stimuli (<1 Hz), the VOR is suppressed by central gaze stabilization systems [10,11].

**Figure 1:**
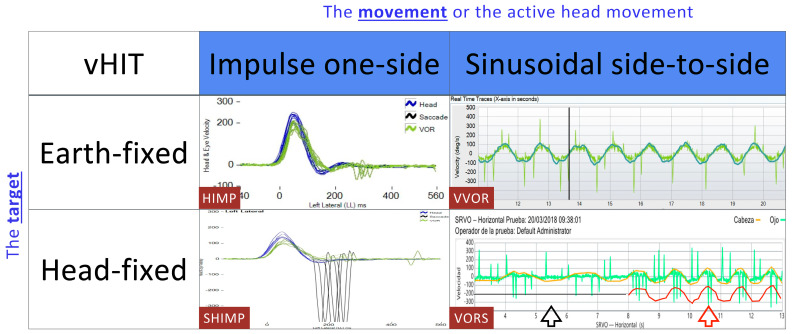
Different vHIT paradigms in relation to movement and gaze fixation points and the recordings obtained in a healthy subject. You can observe the VORS test marked. In black: a stimulation at a reduced frequency is observed, and the ocular response is stable, making the eye movement null and the eyes appear static. In red: the stimulation speed is increased and eye movement begins to be noticeable, with greater gain and more refixation saccades.

High-frequency suppression can be achieved using the SHIMP paradigm [12], while in low-frequency VORS the eye tends to remain static, and its velocity does not match the velocity of the head, potentially displaying a flat, motionless record. In Figure 1, we present the different vHIT paradigms in relation to the movement and the gaze fixation point.

Until recently, VORS interpretation was subjective, depending on the examiner’s impression of the graphical results, as the vHIT does not provide a quantitative measurement method for VORS, limiting it to a qualitative analysis with low applicability for novice examiners [13].

Recently, a new mathematical method has been published for calculating the numerical gains of eye and head velocity, as well as other variables of the VVOR and VORS tests. A computer software implemented in scientific programming environments allows the import of raw VVOR and VORS recordings for quantitative analysis—from now on, quantified VVOR (qVVOR) and quantified VORS (qVORS) (https://github.com/bendermh/VVOR; accessed on 29 July 2024) [14,15]. Subsequent studies have defined the correct methodology for recording and analyzing these paradigms [16,17,18].

The qVORS gain itself has a limited physio-pathological value because, as it involves inhibition, we need to know the actual gain that will be “inhibited” through visual suppression. This gain is obtained using the qVVOR test. Since these are oscillatory (non-impulsive) movements, it is necessary to consider the stimulation frequency. Previous studies have demonstrated the effect of stimulus frequency on gain values [17]. The qVORS paradigm shows a significant reduction in gain as the frequency decreases below 1 Hz [18]. Therefore, to correlate qVVOR and qVORS gains and establish an SI, it is necessary for both paradigms to be performed at a similar stimulation frequency.

Before the vHIT era, attempts were made to establish an SI using the tests available at the time: the caloric and the rotatory tests. Kato theorized about a possible SI calculated using the caloric test, but using the slow phase velocity value, a parameter considered the best representative of the caloric nystagmic response [19,20,21,22]. He distinguished between normal and abnormal suppression, setting the cutoff point at a 60% reduction in slow phase velocity: when the reduction in the slow phase value was less than 60%, it was considered pathological [22]. This procedure was replicated in the rotatory chair testing, where it acquired significant diagnostic value [23,24].

This study aims to determine qVVOR and qVORS gains using a system developed by our research group, generate an SI in healthy subjects, establish normal values, and analyze the influence of demographic variables like age and sex on the visual SI.

## 2. Materials and Methods

A prospective observational clinical study was conducted between March 2018 and March 2023 at the Clínica Universidad de Navarra. A total of 83 subjects were recruited during this period.

Healthy subjects between the ages of 10 and 85 were included. The exclusion criteria were (1) subjects with vestibular pathology, (2) active treatment with drugs affecting the central nervous system, (3) sleep deprivation the night before the examination, (4) cervical pathology limiting or hindering the mobilization required for the vHIT test, (5) eye movements abnormalities, and (6) inability to understand the examiner’s instructions.

The vHIT ICS Impulse equipment (GN Otometrics^®^ Natus Medical Incorporated, Taastrup, Denmark) was used for data collection. The HIMP (head impulse test) recording of the horizontal canals and the SHIMP (head impulse test suppression) test were performed. Subsequently, the qVVOR and qVORS paradigms were conducted. Prior to the study, the examiner was instructed and trained in the correct performance of the HIMP and SHIMP paradigms at different velocities, as well as the qVVOR and qVORS tests.

The horizontal canals were studied using HIMP and SHIMP to verify VOR normal function. The seated subject was asked to look at a point approximately 1 m away and at the same height as their eyes, and the system was calibrated. Fifteen valid and high-quality impulses were obtained for each horizontal canal. If a pathological gain was found in the HIMP (<0.7), the test was interrupted, and the recording was discarded, resulting in the non-inclusion of the patient in the study [12,25].

### 2.1. Recording of Sinusoidal Tests (qVVOR and qVORS)

The methodology for conducting the tests followed the protocols described by Rey-Martínez and Soriano Reixach [14,17]. A predetermined stimulation frequency of 0.75 Hz was established for the qVORS and qVVOR paradigms, and a digital metronome was used to maintain this frequency (45 oscillations per minute). Frequencies below 1 Hz are those that produce the best levels of visual suppression, as only suppression, SP, and OKN are active [7,8], while frequencies above 1 Hz are more influenced by the VOR. Previous studies indicate that exceeding a frequency of 1.4 Hz results in a significant reduction or absence of visual suppression [17]. Before the final recording, preliminary qVVOR and qVORS tests were conducted to ensure the subject understood the procedure.

During the tests, head movements were performed passively: the examiner placed their hands on the subject’s head and began a smooth and continuous movement from side to side (left to right) [14,16]. The guidelines for conducting qVVOR and qVORS are as follows:

- Maintain a smooth and continuous movement.
- Avoid prolonged stops at extreme head positions, aiming for a sinusoidal movement as much as possible.
- Avoid dominance, bias, and asymmetry to the right and left during oscillations.
- Perform oscillations describing a symmetrical arc of about 30–40° amplitude centered at the front.
- Two 17 s recordings were obtained per subject (qVVOR, qVORS).
- During the qVVOR recording, the fixation point was stable on the wall, but during the qVORS, it was displayed from the goggles, and in this test, the examiner repeatedly instructed the subject throughout not to lose sight of the moving laser point on the wall, aiming to achieve a paradigm with cognitive reinforcement according to the work of Soriano Reixach [17].

### 2.2. Mathematical Analysis and Gain Quantification of qVVOR and qVORS

A specific method was developed for the analysis and quantification of the data obtained from qVVOR and qVORS. The foundations of this analysis methodology have previously been published [14].

This analysis method was developed as a specific self-executable computer algorithm and open-access tool called “VVOR” (https://github.com/bendermh/VVOR; accessed on 29 July 2024) for use with MATLAB or OCTAVE computational systems. The developed software is a graphical interface tool that analyzes CSV (comma-separated values) files exported from the vHIT equipment and provides the main mathematical parameters necessary for the description of the qVVOR and qVORS tests [14]. Only the gain and frequency of the qVVOR and qVORS tests were obtained after removing saccades using the Slope regression method according to Rey Martínez, although this method has additional applications.

The first and last seconds of each 17 s recording were removed. The remaining 15 s recordings were divided into three 5 s segments, and according to the work of Soriano Reixach, the central intervals were selected for analysis. Therefore, the analyzed interval was from the 8th to the 13th second [17].

A comparison of the frequencies of the qVVOR and qVORS tests with the expected frequency of 0.75 Hz was performed to determine if there were statistically significant differences using the one-sample Student’s *t*-test.

### 2.3. Calculation of the Suppression Index (SI)

For the calculation of the SI, we would use Demanez’s formula with various modifications, from the caloric and rotational test to the vHIT equipment for the qVVOR and qVORS tests. The SI will be evaluated using the following formula [26,27]:

(1) $$SI(unilateral)=\frac{qVORS\;Gain}{qVVOR\;Gain}100$$

Equation (1): Suppression index.

In this way, a unilateral SI was obtained, considering only the suppression produced by the left or right side separately. The calculation of the unilateral SI is simpler, as it only relates the gain values of the qVVOR on one side to the qVORS on the same side.

To obtain a global or bilateral SI, we adjusted the SI formula, considering gain asymmetry according to the formulas previously used by Matiñó-Soler [28]:

(2) $$Gain\;Asymmetry\;(Asym)=\frac{Minor\;Gain}{Major\;Gain}$$

Equation (2): Gain asymmetry according to Matiñó-Soler. In the numerator, the left–right gain with the lower value, and in the denominator, the higher. Asym: asymmetry of any gain.

If we apply gain asymmetry to the SI formula and consider the average left and right gains of the qVVOR and qVORS, we obtain the formula for the bilateral SI:

(3) $$SI(bilateral)=\frac{qVORS\;Gain\times Asym}{qVVOR\;Gain\times Asym}100$$

Equation (3): SI considering gain asymmetry. Asym: asymmetry of any gain.

## 3. Results

The 83 healthy subjects had a mean age of 44.35 ± 20.51 years, with 41 women (49.4%) and 42 men (50.6%). The age distribution can be found in Table 1.

The gain variables of all tests follow a normal distribution, as calculated by the K–S test. The stimulation frequency and age variables follow a non-parametric distribution, and the sex variable is dichotomous. The gain results for each of the tests performed are presented in Table 2.

### 3.1. Expected Frequency vs. Obtained Frequency (qVVOR and qVORS)

The Wilcoxon signed-rank test for a single sample was used for this comparison, with a reference frequency of 0.75 Hz. In the qVVOR tests, the actual frequency (FrecV) was 0.825 Hz, showing a statistically significant difference (*p* < 0.001) of 0.074 Hz from the expected frequency, while in the qVORS tests, the actual frequency (FrecS) was 0.834 Hz, showing a statistically significant difference (*p* < 0.001) of 0.077 Hz from the expected frequency.

### 3.2. Calculation of the Suppression Index (SI) for Normal Subjects

#### 3.2.1. Calculation of the Unilateral SI

**Figure 2:**
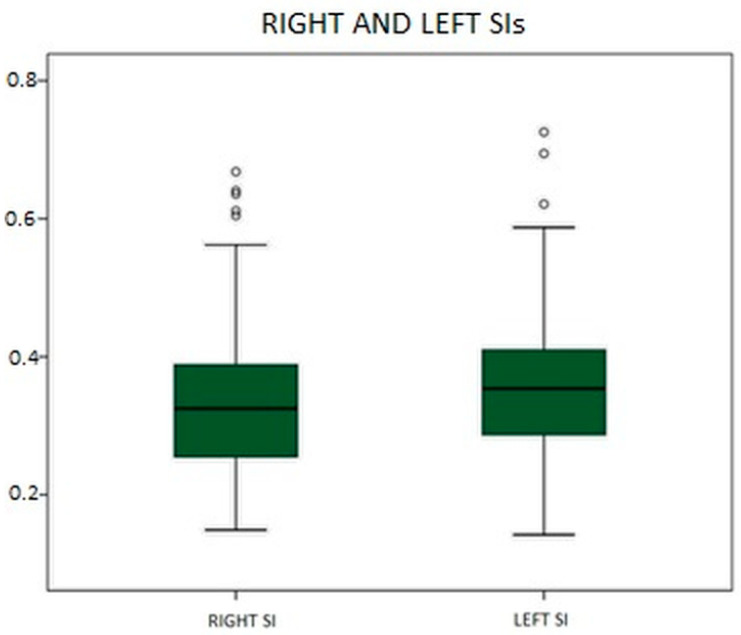
Representation of the distributions of the left and right SIs.

The obtained SIs were 0.363 ± 0.117 for the left side (left SI) and 0.342 ± 0.118 for the right side (right SI). These two new variables in our database followed a normal distribution according to the Kolmogorov–Smirnov test, as they are ratios of two variables with such a distribution. Illustratively, the percentiles of the left and right SIs are presented in Figure 2 and Table 3.

#### 3.2.2. Calculation of the Bilateral SI

The SI formula was corrected using the gain asymmetry calculated with Matiñó’s formula. The bilateral SI has a mean value of 0.295 ± 0.104. The values of the bilateral SI are shown in Table 2.

#### 3.2.3. Calculation of SI Stratified by Sex and Age

##### Differences in SI by Sex

**Figure 3:**
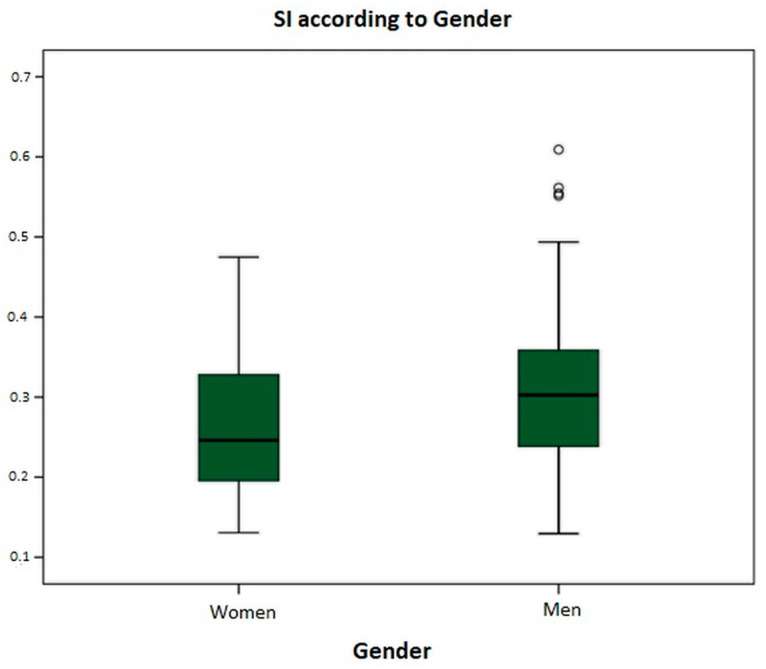
Box plot representing the suppression index divided by gender.

For this analysis, the bilateral SI was used, obtaining an SI value of 0.270 ± 0.092 for women and 0.319 ± 0.111 for men. Figure 3 shows the graphical differences between the SIs for men and women. Comparing both gains using the independent-samples Student’s *t*-test, a mean difference in the SI of 0.049 was found, which is statistically significant (*p* = 0.032). The SI is higher in men.

##### Differences in SI by Age

Similar to the analysis by sex, the bilateral SI was used for this analysis. The previously mentioned age groups were established: ≤20, 21–30, 31–40, 41–50, 51–60, 61–70, 71–80, and >80. The SI values are presented in Table 4.

**Figure 4:**
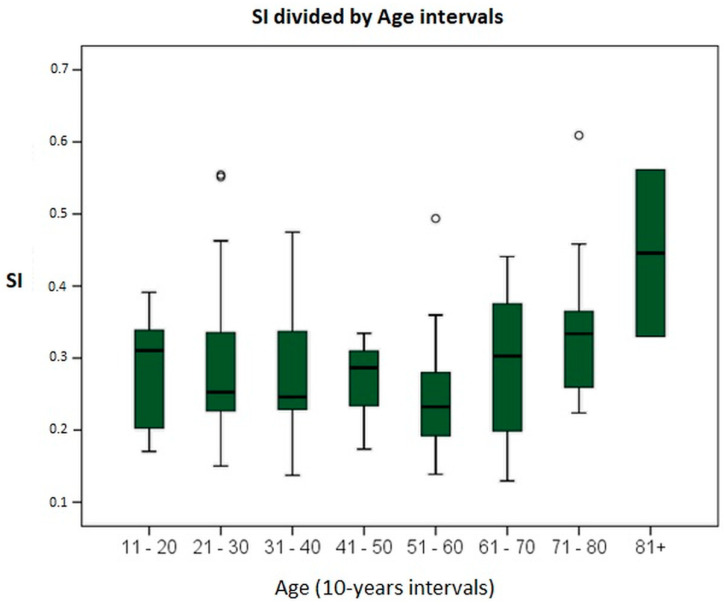
Graph showing SI values in relation to the age of the subjects, grouped in 10-year intervals.

Initially, homogeneity of variances between age groups was demonstrated using Levene’s test, with a statistic of 0.911 (*p* = 0.503). Subsequently, to search for differences in SIs across different age groups, a one-way ANOVA test was performed. No intergroup differences (F = 0.089, *p* = 0.807) or intragroup differences (*p* = 0.324) were observed. Since no differences were found between any of the SIs, post hoc multiple-comparison tests were not performed. Figure 4 shows the distribution obtained in the study, representing the SI by age groups.

Figure 4 shows a trend of an increasing SI with advancing age. For this reason, the age categories were changed, grouping the subjects into two intervals: ≤60 years and >60 years. In our sample, there are 57 subjects aged 60 years or younger and 26 subjects older than 60 years. The mean SI in subjects aged 60 years or younger was 0.281 ± 0.095, and 0.324 ± 0.119 in subjects older than 60 years. To assess the existence of differences, the independent-samples Student’s *t*-test was performed, obtaining equality of variances and a mean difference of −0.042 ± 0.066 (*p* = 0.088), with a trend towards statistical significance.

## 4. Discussion

Since the description in 1988 of the clinical sign of canal paresis, the head impulse test, and earlier in 1979 of the visual–vestibular interaction and visual suppression tests by Halmagyi, otoneurology has entered a new phase in the study of the vestibulo-ocular reflex (VOR) [1,29]. High frame rate video recording has enabled us to track and measure head and eye velocity during the conventional HIMP paradigm of the horizontal canals [30]. The SHIMP paradigm and the examination of the vertical canals were also included in these advancements [31].

The use of custom-made programs for the quantification of saccadic responses with the HITCal software (HITCal 5.3) [15] expanded the evaluation of the VOR to better characterize saccades in cases of vestibular deficit. This system also allowed for a convenient graphical representation of the new video-implemented tests, the VVOR and VORS [16,17]. Following developments allowed for the quantification of numerical gains for these paradigms. A specific methodology for performing both qVVOR and qVORS has also been described [14,18].

The designed program is the key element of our work; it is the innovation that allows for the analysis and comparison of numerical data in addition to graphs. It simplifies the presentation of results for less experienced specialists and may, in the future, enable the establishment of cut-off points between healthy and diseased individuals, potentially becoming a routine implementation in vHIT equipment [14,18]. Currently, any interested researcher can perform these analyses, as the software is free and open access [16].

### 4.1. Sinusoidal Tests: VVOR/VORS

These paradigms are inherently predictable, and the use of a metronome increases the subject’s prediction capability, allowing for close adherence to a desired frequency with an acceptable margin of error. In fact, if we were to eliminate the predictability of the VVOR and VORS tests, we would be performing something similar to HIMP and SHIMP, respectively [32]. Although studies analyzing the graphical interpretations of VVOR and VORS are beginning to emerge [32], our line of research is the only one that numerically addresses VVOR and VORS.

In these tests, error is inevitable, as it is impossible to precisely adjust the head movement to the desired frequency [14]. The examiner responsible for performing the tests was properly trained [33] and followed all aids to maintain a constant frequency: prior training, real-time feedback from the computer, and the use of a metronome [17]. Even in the works of Della Santina [5], it is shown that despite using an automated exploration system, there remains a certain amount of error in the actual frequency of 0.01 Hz [34]. In our study, we report significant differences in actual frequencies of approximately 0.05 Hz, which are not much greater than those recorded by an automated device, and we do not consider them to influence gain results. That is, they are not clinically relevant.

### 4.2. Impulsive vs. Sinusoidal Rotatory Stimulus

The qVVOR and HIMP cannot be the same because they apply completely different stimuli (see Figure 1). We have previously shown that the qVVOR paradigm presents an area under the ROC curve of 0.92 for measuring the VOR gain compared to the HIMP paradigm, and therefore has excellent clinical validity for detecting a vestibular deficit of the horizontal semicircular canal [16,18].

The qVORS and SHIMP are also performed with different stimuli. However, the differences found are more significant, with SHIMP clearly having a higher gain and qVORS having the lowest. It would be interesting for future studies to explore these differences, as there seems to be little difference in gain between the non-suppressed tests (HIMP and qVVOR), while there is a substantial difference in the suppressed tests (SHIMP and qVORS) [18,35], indicating the involvement of different suppression mechanisms.

Halmagyi asserted that to measure true suppression, it is necessary to stimulate with frequencies < 1 Hz [1]. According to this assertion and the gain data in our study, qVORS would represent the true suppression of the VOR. Although the frequencies have been slightly higher than expected, it is estimated that this does not alter the study procedure and the results obtained at all because it has been demonstrated that the desired frequency should be <1 Hz, and despite our deviation, the frequency remains below this value [17].

The two main tests in this study are the qVVOR paradigm and the qVORS paradigm, both of which are visual and vestibular interaction tests. Conceptually, suppression is actually a form of visual tracking, and as a summary we can state that the visual–vestibular interaction produced in the qVVOR measures the VOR plus visual tracking, while the interaction in the qVORS evaluates the VOR minus the visual tracking. It is this latter concept that represents the visual suppression of the VOR.

### 4.3. Suppression Index (SI)

It is necessary to relate the gains of the qVORS and qVVOR to obtain the SI [22]. The main intention of this study is to establish the normal values of the SI for healthy subjects, which should be mathematically calculated by relating the gains of a suppressed test and a non-suppressed test. This SI should represent a reduction in the VOR gain (qVORS) relative to the VOR itself (qVVOR). However, to ensure correct calculation, we must ask if the qVVOR represents the VOR and if the qVORS truly represents its suppression [21].

Previously, it has been demonstrated that the qVVOR represents the VOR with high correlation, showing an area under the ROC curve of 0.92 [16]. The SHIMP paradigm evaluates the suppression of the VOR, either through gain or saccades, and the same is true for the qVORS [32,36].

Therefore, despite being different tests with different results, the HIMP and qVVOR evaluate the VOR, while the SHIMP and qVORS evaluate its suppression. To facilitate differentiation, we have used the terms “impulsive” and “sinusoidal” to define the type of stimulus used for their measurement. Using this same terminology, we could refer to the relative reduction of the HIMP by the SHIMP as the impulsive SI and the reduction of the qVVOR by the qVORS as the sinusoidal SI [12].

The formula representing actual suppression is the quotient between the qVORS and the qVVOR, which we have termed the SI. We consider this index to represent a new paradigm for studying the visual suppression of the VOR.

Healthy subjects exhibit a normal VOR, so the gain measured in the qVVOR is always close to 1. Therefore, when evaluating the mathematical formula of the SI, if the denominator of a quotient is always one, the value obtained is equivalent to the numerator. We present here that the SI and the gain of the qVORS almost overlap and are close to 0.35. One might think that the SI does not provide additional information compared to the qVORS, since identical values are obtained; however, this is not the case, as our study only evaluates healthy subjects with normo-functional VOR. It is in the evaluation of subjects with pathology where differences between the qVORS and SI might be found [22,37].

In 1968, Demanez described suppression and, in his studies using the caloric test, identified a so-called post-caloric nystagmus, representing the suppression of the nystagmus [26]. Later, in 1984, Kato defined the normal caloric SI as a 60% reduction in slow phase velocity. In our study, we obtained a qVVOR gain of 1 and a qVORS gain of 0.35, indicating a reduction slightly greater than 60%. Our study presents results similar to those of Kato and Demanez [22].

When discussing the qVVOR, we are considering two distinct reflexes triggered separately by the right and left labyrinths with their respective stimulations and inhibitions. However, when considering suppression or qVORS, we are theoretically studying the unique function of the central systems and the cerebellum in modulating the eye response [38]. This modulation uniquely affects both labyrinths.

The unilateral SI considers or relates the function of one labyrinth and the central modulation, while the bilateral SI considers both labyrinths and the similar modulation taking place mainly at the cerebellum. If we take labyrinthine function as the reference, we should use two unilateral SIs. Conversely, if we take central suppression as the reference, we should consider a single bilateral SI. It is complex to argue, from a theoretical standpoint, which of the indexes makes more sense.

When stratifying the SI results by sex and age, we used the bilateral SI. Statistically significant differences in the SI were found in relation to the sex of the participants. A bilateral SI of 0.27 ± 0.092 was obtained in women and 0.319 ± 0.111 in men, indicating that the SI is 0.049 lower in women, representing better suppression values. We do not have a consistent hypothesis to explain this finding, so further studies will be conducted to understand it better.

Regarding age differences, as shown in Figure 4, the bilateral SI values remain very similar until the age of 70. Beyond this point, the SI begins to increase, reflecting a reduced suppression capacity in the age intervals of 71–80 (0.344) and >80 (0.446). Matiñó-Soler published the results of the HIMP gain in relation to age, noting a reduction in gain starting at age 70 [28]. If we consider that both the HIMP and qVVOR evaluate the VOR, and that it is the VOR that loses function with age, starting at 70, we can interpret that it is the reduction of the qVVOR, being in the denominator of the SI formula, that changes and alters the SI values.

To corroborate these findings, the sample was divided into two groups: ≤60 years and >60 years. When comparing the means, small differences (0.042) with a trend towards significance were observed. These differences can be evaluated with more powerful statistics in the future when we increase the sample size of subjects over 60 years of age.

The main limitation of this study is that it focuses on healthy subjects and does not include subjects with vestibular pathology. For this reason, it is currently not possible to determine whether the obtained SI is valid or useful for distinguishing between healthy and diseased individuals. In any case, this investigation represents the future direction of this research group. Further studies will be conducted to determine if the qVORS can discriminate between healthy and diseased individuals for central and peripheral pathologies, or if it needs to be complemented with the qVVOR and the SI.

## 5. Conclusions

In conclusion, the sinusoidal tests qVVOR and qVORS are easy to perform; however, achieving the exact stimulation frequency is challenging, even with the use of a metronome. Nonetheless, small variations in stimulation frequency do not alter the gain of the tests. It is possible to establish an SI by relating the gain of the qVORS to that of the qVVOR using Demanez’s formula to obtain a unilateral SI. The values of this SI are 0.342 ± 0.118 for the right side and 0.363 ± 0.117 for the left side. The bilateral SI values are calculated by correcting its formula for gain asymmetry, resulting in a value of 0.295 ± 0.104. The SI is significantly lower in women. There appears to be a trend towards a higher SI with increasing age. Further studies are needed to confirm these findings.

## Figures and Tables

**Table 1:** Distribution of the study population by age ranges. Freq: frequency. %: percentage.

Age	Freq (%)
≤20	8 (4.9)
21–30	19 (11.6)
31–40	9 (5.9)
41–50	11 (6.7)
51–60	10 (6.1)
61–70	15 (9.1)
71–80	9 (5.9)
>80	2 (1.2)

**Table 2:** Gain values of the impulsive tests HIMP, SHIMP, VVOR, and VORS. r: right; l: left.

	Gain	Ranges
HIMPr	1.008 ± 0.099	0.77–1.27
HIMPl	0.940 ± 0.096	0.70–1.37
SHIMPr	0.939 ± 0.116	0.71–1.40
SHIMPl	0.877 ± 0.104	0.65–1.32
qVVORr	0.981 ± 0.070	0.791–1.177
qVVORl	0.978 ± 0.077	0.789–1.204
qVORSr	0.334 ± 0.112	0.140–0.694
qVORSl	0.353 ± 0.110	0.142–0.615

**Table 3:** Percentile values of the different SIs.

Percentile	Left SI	Right SI	Bilateral SI
p5	0.202	0.182	0.141
p10	0.224	0.225	0.181
p25	0.287	0.254	0.224
p50 (Median)	0.354	0.325	0.283
p75	0.412	0.390	0.348
p90	0.530	0.529	0.451
p95	0.584	0.610	0.540

**Table 4:** Distribution of bilateral SI by age, grouped in 10-year intervals.

Age (Ranges)	SI
≤20	0.283 ± 0.081
21–30	0.299 ± 0.116
31–40	0.279 ± 0.101
41–50	0.273 ± 0.052
51–60	0.259 ± 0.103
61–70	0.295 ± 0.107
71–80	0.344 ± 0.125
>80	0.446 ± 0.163

## Data Availability

Data available on request due to restrictions.
